# Is Hume's Law a valid argument against empirical bioethics?

**DOI:** 10.1111/bioe.13393

**Published:** 2025-01-25

**Authors:** Paolo Corsico

**Affiliations:** ^1^ School of Public and Population Health Institute for Bioethics and Health Humanities, The University of Texas Medical Branch Galveston Texas USA; ^2^ Alliance Manchester Business School Manchester Institute of Innovation Research, The University of Manchester Manchester UK

**Keywords:** bioethics, empirical bioethics, Hume's Law, Is‐Ought problem, non‐cognitivism

## Abstract

If “no ought from is,” how can bioethics be empirical? Despite the widespread recognition that we can integrate empirical and normative, Hume's Law is still often claimed to pose logical limitations to empirical bioethics. Is Hume's Law a valid argument against empirical bioethics? I argue that we have reasons to answer no. First, I outline and reject two unverified assumptions: that Hume’ s Law, the fact‐value distinction, and the naturalistic fallacy are roughly the same thing, and that Hume's Law is an undisputed meta‐ethical principle which dictates how to formulate normative statements. I then show how the interpretation of Hume's Law as establishing a logical gulf between facts and morality—rather than as clarifying the logical rules of normative argumentation—is dependent upon a non‐cognitivist interpretation of the Is‐Ought problem. I argue that the version of Hume's Law that stems from ethical non‐cognitivism is what is problematic for empirical bioethics. However, other interpretations are possible. We have two reasons to reject the thesis that Hume's Law is an argument against empirical bioethics. First, conflating meta‐ethics and applied ethics is problematic. Second, a non‐cognitivist interpretation of Hume's Law is likely to constitute an argument not only against empirical bioethics, but against all bioethics that claims to be situated within ethical cognitivism, be it empirical or philosophical. Lastly, I present two meta‐ethical postulates shared by empirical and philosophical bioethics. I call them: (1) the “bridge” postulate and (2) the “ethical cognitivism” postulate.

## 1 INTRODUCTION

Discussions concerning the epistemological status of empirical bioethics often cite the Is‐Ought problem stemming from David Hume's *A Treatise of Human Nature*—also known as Hume's Law—as potentially impeding on the use of empirical data in normative theory and therefore as a potential argument against empirical bioethics.^1^ Briefly, if “no ought from is,” then using empirical data in normative theory as empirical bioethics attempts to do is a doomed exercise. By establishing logical limitations to how what “is” can be used to argue what “ought to be,” Hume's Law would allegedly establish logical limitations to the use of empirical methods in bioethics. The debate has been ongoing for decades. Despite attempts to settle the issue in favor of integration of is and ought^2^ as well as recent consensus efforts that indicate that integration may not only be possible but even *necessary*,^3^ the spectre of Hume's Law keeps haunting empirical bioethics.

In this article, I address the question: Is Hume's Law a valid argument against empirical bioethics? I argue that we have reasons to reject Hume's Law as an argument against empirical bioethics. These reasons relate to how we interpret Hume's Law and its role in 20th‐century moral philosophy. An important contention of this article is that the reason the Is‐Ought problem has influenced how we approach empirical bioethics is as historical as it is theoretical. The way we approach empirical bioethics reflects the historical evolution of Western moral philosophy in the 20th century and the theoretical issues that bioethics inherited from that season of moral philosophy.

It is important to clarify what this article is *not* about. This article is not about how to combine the empirical and the normative in empirical bioethics. The widespread acceptance which the argument that such combination is possible and worthwhile has received in the past 15 years is testified by the flourishing of literature on the matter.^4^ Nor is this article about the broader issue of the role that empirical research can play in bioethics; even more has been written, and for longer, about this issue.^5^ This article is also not about two distinct issues which are often conflated with or addressed alongside Hume's Law: the naturalistic fallacy and the fact‐value distinction. I do touch on both issues. However, I do not discuss whether they constitute valid arguments against empirical bioethics. Most importantly, this article does not attempt to answer the Is‐Ought question or to take a definitive stance on the implications of Hume's Law for normative analysis. The article only attempts to evaluate the theoretical and historical significance of Hume's Law in relation to the epistemological foundations of empirical bioethics.

In the last section of the article, I introduce two meta‐ethical postulates which I argue are shared by empirical and philosophical bioethics. They are postulate 1: A bridge between facts and values is both (i) possible and (ii) foundational to bioethics; the nature of this bridge remains open to discussion. I call this the “bridge postulate.” And postulate 2: Unless explicitly stated, bioethics is meta‐ethically situated within ethical cognitivism, understood as the argument that ethical statements are truth‐apt and that knowledge is possible in ethics. I call this the “ethical cognitivism” postulate.

## 2 UNVERIFIED ASSUMPTIONS: HUME'S LAW AND EMPIRICAL BIOETHICS

Discussions of Hume's Law often begin with reporting the famous Is‐Ought passage from Book III, Part I of Hume's *A Treatise of Human Nature*. Scholars then usually proceed to provide some interpretation of the passage and to draw some conclusions based on that interpretation. The passage is easily accessible in much of the literature on the matter, so I do not report it here. I also believe that it is more useful to start from a specific interpretation of the Is‐Ought passage. John Searle writes:

It is often said that one cannot derive an 'ought' from an 'is'. This thesis, which comes from a famous passage in Hume's *Treatise*, while not as clear as it might be, is at least clear in broad outline: there is a class of statements of fact which is logically distinct from a class of statements of value. No set of statements of fact by themselves entails any statement of value. Put in more contemporary terminology, no set of *descriptive* statements can entail an *evaluative* statement without the addition of at least one evaluative premise. To believe otherwise is to commit what has been called the naturalistic fallacy.^6^

Searle's outline of the Is‐Ought problem presents clearly and concisely the issues at stake when 20th century moral philosophers interrogated the Is‐Ought passage beyond the historical analysis of what Hume intended to say. It is an interesting starting point because it explicitly puts together the Is‐Ought problem, the fact‐value distinction, and the naturalistic fallacy. Those issues are distinct although interconnected. Conflating them has been a recurring feature of contemporary moral philosophy and bioethics, and this conflation may have contributed to generating confusion on the matter.

It is difficult to say when the Is‐Ought problem was first discussed in relation to bioethics. A reason might be that the Is‐Ought problem was perceived as a central problem of moral philosophy—or *the* central problem, as reads the title of a volume edited by W. D. Hudson—in the 1960s, the same years when bioethics was being established as an independent discipline.^7^ Robert Veatch mentioned the Is‐Ought problem in relation to bioethics as early as 1973.^8^ John Harris attempted to “put empirical studies in their place” by providing a strong refutation of the use of empirical data in bioethics in 2004. He accused much contemporary bioethics of committing an “empiricalistic fallacy.”^9^ Beyond the validity of his argument, Harris’ conflation of Hume's Law and the naturalistic fallacy remains problematic.^10^ In their famous article on the empirical turn in bioethics, Borry and colleagues mentioned the Is‐Ought problem as one of the reasons why empirical approaches to bioethics have not flourished.^11^ They also conflated Hume's Law and the naturalistic fallacy.^12^ Ives and Draper discussed the significance of the Is‐Ought problem in relation to the “social science critique” of bioethics.^13^ They noted the frequent conflation of the Is‐Ought problem with the fact‐value distinction and the naturalistic fallacy, conflation they admit committing themselves. De Vries and Gordijn addressed these three issues and called them empirical ethics’ “alleged meta‐ethical fallacies.”^14^ They showed how these meta‐ethical fallacies are distinct even though interrelated. More recently, John McMillan thoroughly engaged with Hume's Law in relation to empirical bioethics, even though he claims to be addressing the fact‐value distinction.^15^

A first unverified assumption I wish to highlight is the argument that Hume's Law, the fact‐value distinction, and the naturalistic fallacy are (roughly) the same thing. They are not. De Vries and Gordijn explain this very well.^16^ “Naturalistic fallacy” is an expression introduced by G.E. Moore in his *Principia Ethica*. It broadly means that it is a logical fallacy to define “good” in terms of natural properties. It represents a meta‐ethical refutation of ethical naturalism. The notion of “fact‐value distinction,” as De Vries and Gordijn show, does not point to a problem or to a fallacy but rather to the view that facts and values are, indeed, distinct. This distinction may be taken to mean many things: that factual statements and value statements have different truth‐apteness or that scientific facts and ethical values are distinct, and science is value‐free. This second view—the positivist view that science is value‐free—has historically been contested by social scientists including proponents of the social science critique of bioethics such as Adam Hedgecoe.^17^ The important point here is that, even though “fact‐value distinction” has often been understood as a synonym of Hume's Law, the two notions are not synonyms. So, what exactly is Hume's Law?

Before addressing this question, I wish to dismiss a second unverified assumption: the idea that Hume's Law is an undisputed meta‐ethical principle which when translated into empirical bioethics would dictate what we can or cannot do when formulating normative statements. This is historically and theoretically inaccurate. A reason why philosophers talk about the Is‐Ought *problem* is that the relationship between “is” and “ought” or of descriptive statements and evaluative statements is problematic. It is an area of meta‐ethical disagreement. The Is‐Ought problem was popular among philosophers until the late 1960s—yet this does not mean that there was agreement on what the Is‐Ought problem meant or on what Hume's Law dictated. If anything, the opposite is true. Positioning oneself within this debate often meant defining one's own meta‐ethical theory in relation to terms such as “good,” “value,” and “morality.” Unpacking some of the history of the Is‐Ought problem may help us to figure out what we can say of Hume's Law and empirical bioethics.

## 3 WHAT EXACTLY IS HUME'S LAW?

The Is‐Ought problem is the question about the relationship between “is” statements and “ought” statements or descriptive statements and evaluative statements. It has been shaped by discussions of the Is‐Ought passage in the *Treatise*. Even though until now I have used the expressions “Is‐Ought problem” and “Hume's Law” as synonyms, they are not, to be more accurate, synonyms. They do refer to the same issue, unlike the expressions “naturalistic fallacy” and “fact‐value distinction.” However, “Is‐Ought problem” indicates the problematic interpretation of the Is‐Ought passage. “Hume's Law” indicates a certain *stance* on the Is‐Ought problem, a certain interpretation of it which indicates what we can or cannot do when formulating evaluative statements. I am not sure of who first talked about “Hume's Law.” The first mention of the expression I can find is by R. M. Hare in 1955.^18^ Hare does not define what he means by Humes’ Law in this passage. A synonym of “Hume's Law” often found in the literature is “Hume's Guillotine.” I am rather confident this expression was invented by Max Black.^19^ If it is relatively easy to define what “Is‐Ought problem” means, defining “Hume's Law” is more difficult as any definition of Hume's Law is likely to contain some interpretation of the Is‐Ought problem.

Hume's Law can, loosely, indicate the argument that it is not possible to pass from “is” or descriptive statements to “ought” or evaluative statements; or that it is not possible to pass from *purely* “is” statements to “ought” statements. Every definition of Hume's Law comes with its own set of entailments and criticism. In the bioethics literature, Hume's Law appears to be understood as a *logical* principle. Ives and Draper recognize the logical nature of Hume's Law.^20^ De Vries and Gordijn affirm that there are two ways of understanding this logical principle: “[…] It either states (1) that no moral conclusions can be derived from nonmoral premises alone or (2) that no evaluative conclusion can be derived from descriptive premises alone.”^21^ The interpretation of Hume's Law as a logical principle and the definitions given by De Vries and Gordijn are consistent with the historical debate of the Is‐Ought problem. Alasdair MacIntyre affirmed: “The standard interpretation of this passage takes Hume to be asserting here that no set of non‐moral premises can entail a moral conclusion.”^22^ I have already reported Searle's definition.^23^ Another fundamental definition of Hume's Law is provided by Hare: “No imperative conclusion can be validly drawn from a set of premisses which does not contain at least one imperative.”^24^ Hare does not use the expression Hume's Law in this passage. However, he states that this is his interpretation of the Is‐Ought passage.

If Hume's Law is *only* a logical principle, then all it does is remind us that we must include some moral, evaluative, or imperative premises in our argument if we wish to draw moral, evaluative, or imperative conclusions. All Hume's Law does is to tell us how to argue well. De Vries and Gordijn conclude so.^25^ Ives and Draper seem to argue the same.^26^ McMillan comments: “If that's all he [Hume] means here, then it is a helpful reminder to all involved in bioethics that they should check the ethical premises that they're using to see whether they imply the normative conclusions that have been draw.”^27^ This is an interesting interpretation of Hume's Law. So understood, Hume's Law would be a logical principle that can be integrated easily within empirical bioethics. It would not constitute an argument *against* empirical bioethics.

However, if Hume's Law is only a logical principle and this is all it does, why all the fuss? Why was the Is‐Ought problem the central problem of moral philosophy in the 1960s? McMillan recognizes this fact. He shows that unfortunately things are more complicated. He discusses how a certain interpretation of the Is‐Ought problem in connection with Hume's epistemology and emotivism can be—and historically has been—used to express criticism about the possibility of grounding normative claims.^28^

Here is where I believe delving into the history of the Is‐Ought problem is essential to evaluate whether Hume's Law can be considered a valid argument against empirical bioethics and to begin to understand why it recursively comes back to haunt it. The volume edited by Hudson in 1969 is an invaluable tool for this task.^29^ Hudson's introduction shows how the crux of the matter is not whether the Is‐Ought problem is only a logical problem or whether Hume's Law is only a logical principle. The real issue is what Hudson calls the *logical gulf* between “is” and “ought.”^30^ A tradition of philosophers that passes from logical positivism and includes A. J. Ayer, Hare, and P. H. Nowell‐Smith understands factual or descriptive statements as being of a completely different nature compared to moral, evaluative, or imperative statements. For these philosophers, what opens between facts and morality is a logical gulf. Ayer's version of logical positivism influenced a generation of analytic philosophers.^31^ If Ayer's critique of ethics resembles that of Hume, Hare and Nowell‐Smith were influential in pointing to the Is‐Ought passage as the foundation of their claim of a logical gulf between facts and morality. As Charles Pigden says:

[…] many philosophers, (notably Hare and Nowell‐Smith) have read their own non‐cognitivism back into Hume, and gone on to contend that the *point* of this famous passage is to distinguish between two logical or semantic categories: propositions on the one hand, and value‐judgements – variously understood as exclamations or orders – on the other.^32^

There is much to unpack in this quote from Pigden, most importantly the issue of non‐cognitivism. I will do so in the next section. Here, it is important to affirm that the relevance of the Is‐Ought problem in the 1950s and 1960s depends on the issue of the logical gulf between facts and morality, which philosophers such as Hare took to mean *not* that Hume's Law tells how to argue, but that Hume's Law dictates what we can or cannot do in normative reasoning.

Following Hudson—and his volume is structured accordingly—most philosophers that discussed the Is‐Ought problem sat on either side of the following fence. They either argued that (1) “is” and “ought” are bridgeable, or that (2) “is” and “ought” are unbridgeable. Among those who argued for (2) — Hare is likely the most famous supporter of this argument—philosophers either argued that (a) “ought” cannot be *reduced to* “is” or that (b) “ought” cannot be *derived from* “is.”^33^ It is not common for those who question the epistemological foundations of empirical bioethics to discuss argument (2)(a), that “is” and “ought” are unbridgeable because “ought” can/cannot be reduced to is.^34^ I believe we can safely disregard this discussion here. However, argument (2)(b), that “is” and “ought” are unbridgeable and that “ought” cannot be derived from “is,” is a good approximation of the interpretation of Hume's Law which sees it as an argument against empirical bioethics. (2)(b) is primarily linked to, and we may also say showcased by, Hare's version of ethical non‐cognitivism and his formulation of universal prescriptivism. Let us see what the issue of the (un)bridgeable gulf between facts and morals can tell us in relation to the question of whether Hume's Law is a valid argument against empirical bioethics.

## 4 IS HUME'S LAW A VALID ARGUMENT AGAINST EMPIRICAL BIOETHICS?

I can now address the question directly: Is Hume's Law a valid argument against empirical bioethics? First, who argues so? In 1993, Baruch Brody summarized the issue in the clearest way:

Ethics is, after all, a normative inquiry; how then can empirical research contribute to it? David Hume's famous is—ought gap (the claim that you cannot derive a moral ought‐conclusion from factual is‐premises) argues that it cannot.^35^

Interestingly, Brody puts this claim in the introduction to his article and then spends much of it assessing the role of empirical research in bioethics. This is a common *modus operandi* in the literature. Scholars usually begin by claiming that Hume's Law is or may be an argument against empirical bioethics. They then go on to either discuss the issue or consider ways in which empirical research can contribute to normative reasoning and empirical bioethics. Most of the literature cited in this article follows this recipe.^36^ Most articles I cited here begin with the *assumption* that Hume's Law may be an argument against empirical bioethics; few of them end up claiming that it is. I can only indicate one author who vigorously claimed that the Is‐Ought passage prevents us from doing empirical research in bioethics, and that is John Harris.^37^ Yet, Hume's Law keeps reappearing in the bioethics literature as one of the arguments against which bioethicists must justify their attempt to integrate empirical and normative. Let us look at what the interpretation of Hume's Law as establishing an (un)bridgeable gulf between facts and morality—and this argument's links to ethical non‐cognitivism—can tell us about this matter.

### 4.1 Hume's Law and non‐cognitivism in ethics

I believe McMillan, De Vries and Gordijn, and Ives and Draper are right: Hume's Law understood as a logical principle which tells us how to formulate a normative argument does not pose logical challenges to empirical bioethics. However, Hume's Law understood as establishing an unbridgeable gulf between facts and morality does. This interpretation of Hume's Law is logically connected to a certain version of ethical non‐cognitivism.

Ethical non‐cognitivism is the meta‐ethical argument that ethical statements—be they moral, evaluative, or imperative—are not truth‐apt and therefore knowledge is not possible in ethics. Ethical non‐cognitivism has its roots in the critique of ethics formulated by logical positivism. In the analytic tradition, this critique was most notably formulated by Ayer in *Language, Truth and Logic*.^38^ Ayer's version of logical positivism restricted knowledge to analytic propositions, which are tautologies and the object of logic and mathematics, and factual statements, which are hypotheses verifiable by empirical observation.^39^ For Ayer, ethics is outside the domain of knowledge as ethical statements cannot be said to be true or false. They are either expressions of emotions or prescriptions. Hence, non‐cognitivism. Moral philosophy is reduced to the analysis of moral language.

Hare's non‐cognitivism, which was influenced by Ayer's, is what is relevant here. Hare's analysis of moral language took him to formulate *universal prescriptivism*: a philosophical theory that claims that ethical statements are not truth‐apt, as they are imperative prescriptions with a claim to universalizability.^40^ It is within this context that we must understand Hare's interpretation of Hume's Law and the discussion about the (un)bridgeable gulf between facts and morals. Hare's interpretation of Hume's Law is non‐cognitivist. The gulf between facts and morals opens because, according to Hare, Hume was the first philosopher to recognize that “is” statements and “ought” statements are of a completely different nature and cannot be bridged.

Regardless of whether Hare's interpretation of Hume's Law is correct, it was a popular one and so remained in certain philosophical circles.^41^ Hare later published in bioethics.^42^ However, it is also a fact that most scholars who argued that “is” and “ought” are bridgeable can be classified as ethical cognitivists. It is clear, as testified by the volume edited by Hudson, that there existed numerous cognitivist interpretation of the Is‐Ought problem. MacIntyre, Black, Searle, Anscombe, and Foot all presented interpretations of it that sit within ethical cognitivism. Two decades later, Pigden affirmed that the fact that Hare and Nowell‐Smith “read their own non‐cognitivism back into Hume” and understood factual and moral statements as of a completely different kind is what “[…] breaks the inferential bridge between Is and Ought.”^43^

This is my central claim: We *do not have* to adopt a non‐cognitivist interpretation of the Is‐Ought problem—one that claims that Hume's Law opens an unbridgeable gulf between facts and morality, between “is” statements and “ought” statements. Other interpretations of the Is‐Ought problem exist. Most scholars who argued in favor of a bridge between “is” and “ought” adopted a cognitivist meta‐ethical position.

### 4.2 Reasons to answer no

We have two reasons to answer “no” to the question, is Hume's Law a valid argument against empirical bioethics?

The first reason is theoretically weaker than the second, but methodologically important. Regardless of which interpretation of Hume's Law we consider the most convincing—*even* if this is a non‐cognitivist one—Hume's Law and the Is‐Ought problem are situated at the level of meta‐ethics. Bioethics is a branch of applied ethics. Conflating the two levels of abstraction does not seem the best way to solve issues at each level. We can consider meta‐ethics an area of ethics alongside normative ethics and applied ethics. If meta‐ethics is concerned with the nature of moral language, normative ethics with what we ought to do, and applied ethics with what we ought to do in certain situations, bioethics is a branch of applied ethics. These disciplines are not situated in silos as they question each other. Yet, there is a considerable theoretical distance between meta‐ethics as an area of ethics that emerged from analytic philosophy and bioethics as a branch of applied ethics that emerged as a response to moral issues posed by technological development.

Conflating meta‐ethics and bioethics, even though not logically fallacious, is at least methodologically problematic. The Is‐Ought problem concerns the logical relationships between statements, not how we should solve moral issues in biomedicine. Cognitivist interpretations of Hume's Law discuss what we can *know* of morality; non‐cognitivist ones what we can *say* about moral language. More importantly, non‐cognitivist interpretations of Hume's Law question the nature of moral theories in normative ethics such as utilitarianism, deontology, and virtue ethics, and therefore their use in applied ethics. This fact has implications for *both* empirical and philosophical bioethics. It leads me to my second reason.

The second reason is theoretically stronger. McMillan grasps it.^44^ It involves addressing bioethics’ meta‐ethical grounding, as I do in this article—going against the advice I gave in outlining my first reason. If a non‐cognitivist interpretation of the Is‐Ought problem and of Hume's Law is what is problematic for empirical bioethics, we are not bound to adopting it. If we must address bioethics’ meta‐ethical grounding in relation to the Is‐Ought problem, we are better off rejecting ethical non‐cognitivism. Bioethicists might not be best placed to say a definitive word about Hume's Law, but it seems wise for bioethics as a discipline to reject a non‐cognitivist interpretation of it.

Not only would a non‐cognitivist interpretation of Hume's Law, which states that we can never bridge “is” and “ought” because ethical statements are not truth‐apt, be an argument against empirical bioethics. It would be an argument against *all* bioethics that claims to be situated within ethical cognitivism, be it empirical or philosophical. Most literature has failed to grasp this point. A non‐cognitivist interpretation of Hume's Law is likely to constitute an argument against philosophical bioethics as much as an argument against empirical bioethics. If Hume's Law entails ethical non‐cognitivism, then most philosophical bioethics as we practice it is doomed.^45^ Most philosophical bioethics seems to stand on cognitivist grounds.

Of course, Hume is not Ayer, and Ayer is not Hare. However, Hume's emotivism, Ayer's logical positivism, and Hare's non‐cognitivism belong to a philosophical tradition that questions the role of knowledge in formulating normative claims. This tradition's critique has often been directed toward moral theories in Western philosophy. Hume, Ayer, and Hare were all, albeit in very different ways, critiquing philosophical moral theories. Yet, philosophical moral theories constitute the backbone of philosophical bioethics. Using non‐cognitivist arguments to contend that bioethics can only be philosophical seems paradoxical. How many philosophical approaches to bioethics could claim to be situated within ethical non‐cognitivism? Are consequentialist and utilitarian approaches to bioethics non‐cognitivist? Are deontology, virtue ethics, or care ethics? What about principlism, justice theories, and feminist bioethics? All these approaches seem to share the cognitivist assumption that bioethics is a form of knowledge. They ground normative claims on statements of fact, such as who counts as a person, what sentience is, or the appropriate criterion to establish death. They use logical argumentation to bridge statements of fact and statements of value to produce normative claims, which in turn have a claim to constitute knowledge.

There are historical reasons why bioethicists have been concerned by the Is‐Ought problem and have sometimes conflated it with non‐cognitivist interpretations of Hume's Law. Put simply, I believe this happen(ed) because analytic philosophy and meta‐ethics were dominant approaches to moral philosophy in the first half of the 20th century and contributed to the birth of bioethics as an independent discipline.^46^ This historical fact does not entail that we should keep accepting their postulates.

## 5 TWO META‐ETHICAL POSTULATES OF (EMPIRICAL) BIOETHICS

Rejecting a non‐cognitivist interpretation of the Is‐Ought problem—and the subsequent interpretation of Hume's Law as posing logical limitations to (empirical) bioethics—gives us sufficient grounds to formulate two meta‐ethical postulates of bioethics, be it empirical or philosophical:

- Postulate 1: A bridge between facts and values is both (i) possible and (ii) foundational to bioethics; the nature of this bridge remains open to discussion.
- Postulate 2: Unless explicitly stated, bioethics is meta‐ethically situated within ethical cognitivism, understood as the argument that ethical statements are truth‐apt and that knowledge is possible in ethics.

A postulate in philosophy can be considered the equivalent of an axiom in mathematics or logic. It is a principle that is *assumed* to be valid, as it serves as the foundation for further argumentation. I call the two principles above “meta‐ethical postulates” to indicate that, even though they remain debatable at the level of meta‐ethics, they are foundational to bioethics as a branch of applied ethics and are therefore embedded in the way we think of bioethics as a discipline, regardless of whether we conduct philosophical or empirical bioethics. As postulates, they are assumed and “posited.” They must be justified—and I have tried to do so in this paper—but they do not need theoretical argumentation.

I call postulate 1 the “bridge postulate.” It is reminiscent of Van Rensselaer Potter's idea of bioethics as a “bridge” to the future and between ethical values and biological facts.^47^ It is somehow ironic that bioethics was born as a discipline that would bridge facts and values, yet philosophers have criticized that very aspiration in empirical bioethics. We can certainly discuss the nature of the bridge, but it does not seem wise to question its existence. I call postulate 2 the “ethical cognitivism” postulate. The qualifier “unless explicitly stated” means that it is surely possible to develop non‐cognitivist approaches to bioethics, which would endorse a non‐cognitivist interpretation of Hume's Law and therefore reject any bridge between facts and morality. Yet, this is not the common way we think of bioethics. We have reasons to argue that most approaches to bioethics, be they empirical or philosophical, stand on cognitivist grounds and claim, surely in very disparate ways, to be contributing to knowledge.

## 6 CONCLUSIONS

Why have bioethicists conflated Hume's Law, the fact‐value distinction, and the naturalistic fallacy, as well as overlooked the philosophical implications of non‐cognitivist interpretations of the Is‐Ought problem for bioethics? The historical legacy of analytic philosophy and meta‐ethics surely played a role. We can speculate that disciplinary boundaries and funding structures in bioethics may have also played a role. The use of the Is‐Ought problem and Hume's Law to declare empirical research inadmissible in bioethics points to the sometimes troubled relationship between philosophy and the social sciences in the everyday life of bioethics. But this is a story to be investigated elsewhere.

If we understand Hume's Law to be a logical principle which provides rules on how to formulate a normative argument, then Hume's Law is not an argument against empirical bioethics. If we understand Hume's Law as establishing a logical gulf between facts and morality, then we have reasons to reject the non‐cognitivist framework which provides the theoretical foundations of such interpretation as most bioethics, be it empirical or philosophical, seems to claim to be situated within ethical cognitivism—unless stated otherwise. In this second case, we also have reasons to argue that Hume's Law is not a valid argument against empirical bioethics. We are likely justified in affirming that a bridge between facts and morality is possible in philosophical and empirical bioethics, and that bioethics is, most times, a form of knowledge. This of course says nothing about whether the naturalistic fallacy or the fact‐value distinction are valid arguments against empirical bioethics. It says little about *whether* or *how* we can integrate empirical and normative in empirical bioethics. However, it supports the claim that we should stop invoking Hume's Law as a reason why we cannot, or should not, conduct empirical bioethics.

## Footnotes

1 For the Is‐Ought passage, see Hume, D. *A Treatise of Human Nature*. Book III: Of Morals, Part I: Of Virtue and Vice In General, Section I: Moral Distinctions Not Deriv'D From Reason.

2 Kon, A. A. (2009). It is settled: The ‘Is’ can (and should) inform the ‘Ought’! *The American Journal of Bioethics*, *9*(6–7), W4–W6. https://doi.org/10.1080/15265160902966902

3 Ives, J., Dunn, M., Molewijk, B., Schildmann, J., Bærøe, K., Frith, L., Huxtable, R., Landeweer, E., Mertz, M., Provoost, V., Rid, A., Salloch, S., Sheehan, M., Strech, D., de Vries, M., & Widdershoven, G. (2018). Standards of practice in empirical bioethics research: Towards a consensus. *BMC Medical Ethics*, *19*(1), 68. https://doi.org/10.1186/s12910-018-0304-3

4 Davies, R., Ives, J., & Dunn, M. (2015). A systematic review of empirical bioethics methodologies. *BMC Medical Ethics*, *16*(1), 15. https://doi.org/10.1186/s12910-015-0010-3.

5 Brody, B. A. (1993). Assessing empirical research in bioethics. *Theoretical Medicine*, *14*(3), 211–219. https://doi.org/10.1007/BF00995163; De Vries, R. (2004). How can we help? From “sociology in” to “sociology of” bioethics. *Journal of Law, Medicine & Ethics*, *32*(2), 279–292. https://doi.org/10.1111/j.1748-720X.2004.tb00475.x; Holm, S., & Jonas, M.F. (Eds.). (2004). *Engaging the world: The use of empirical research in bioethics and the regulation of biotechnology*. IOS Press; Solomon, M. Z. (2005). Realizing bioethics’ goals in practice: Ten ways “Is” can help “Ought”. *Hastings Center Report*, *35*(4), 40–47. https://doi.org/10.1353/hcr.2005.0048; Kon, A. A. (2009). The role of empirical research in bioethics. *The American Journal of Bioethics*, *9*(6–7), 59–65. https://doi.org/10.1080/15265160902874320.

6 Searle, J. R. (1969). How to derive ‘ought’ from ‘is’. In W. D. Hudson (Ed.), *The Is/Ought question. A collection of papers on the central problem in moral philosophy* (pp. 120–134). MacMillan and Co LTD, p. 120.

7 Hudson, W. D. (Ed.). (1969). *The Is/Ought question. A collection of papers on the central problem in moral philosophy*. MacMillan and Co LTD.

8 Veatch, R. M. (1973). Does ethics have an empirical basis? *The Hastings Center Studies*, *1*(1), 50–65. https://doi.org/10.2307/3527473

9 Harris, J. (2004). Putting empirical studies in their place. In S. Holm & M. F. Jonas (Eds.), *Engaging the world: The use of empirical research in bioethics and the regulation of biotechnology* (pp. 18–27). IOS Press.

10 Harris begins his essay by citing: “David Hume's famous articulation of the naturalistic fallacy […]”, Ibid: 18.

11 Borry, P., Schotsmans, P., & Dierickx, K. (2005). The birth of the empirical turn in bioethics. *Bioethics*, *19*(1), 49–71. https://doi.org/10.1111/j.1467-8519.2005.00424.x

12 They write: “According to most authors, this fundamental distinction [the Is‐Ought distinction] stems from a small paragraph of David Hume's *Treatise of Human Nature* (1740), and is traditionally called the naturalistic fallacy,” Ibid: 60.

13 Ives, J., & Draper, H. (2009). Appropriate methodologies for empirical bioethics: It's all relative. *Bioethics*, *23*(4), 249–258. https://doi.org/10.1111/j.1467-8519.2009.01715.x

14 De Vries, R., & Gordijn, B. (2009). Empirical ethics and its alleged meta‐ethical fallacies. *Bioethics*, *23*(4), 193–201. https://doi.org/10.1111/j.1467-8519.2009.01710.x

15 McMillan, J. (2018). The fact/value spectre. In *The methods of bioethics: An essay in meta bioethics* (pp. 71–83). Oxford University Press; McMillan, J. (2016). Empirical bioethics and the fact/value distinction. In J. Ives, M. Dunn, & A. Cribb (Eds.), *Empirical bioethics: Theoretical and practical perspectives* (pp. 17–32). Cambridge University Press.

16 De Vries & Gordijn, op. cit. note 14, pp. 193–201.

17 Ives & Draper, op. cit. note 13, p. 253.

18 Hare, R. M. (1955). XIII.—Universalisability. *Proceedings of the Aristotelian Society, 55*(1), 295–312. https://doi.org/10.1093/aristotelian/55.1.295

19 Black, M. (1969). The gap between ‘is’ and ‘should’. In W. D. Hudson (Ed.), *The Is/Ought question. A collection of papers on the central problem in moral philosophy*. (pp. 99–113). MacMillan and Co LTD.

20 Ives & Draper, op. cit. note 13, pp. 249–258.

21 De Vries & Gordijn, op. cit. note 14, p. 196.

22 MacIntyre, A.C. (1969). Hume on ‘is’ and ‘ought’. In W. D. Hudson (Ed.), *The Is/Ought question. A collection of papers on the central problem in moral philosophy* (pp. 35–50). MacMillan and Co LTD, p. 36.

23 Searle, op. cit. note 6, p. 120.

24 Hare, R. M. (1952). *The language of morals* (p. 28). Oxford University Press.

25 De Vries & Gordijn, op. cit. note 14, p. 200.

26 Ives & Draper, op. cit. note 13, p. 253.

27 McMillan (2018), op. cit. note 15, p. 73.

28 McMillan (2016), op. cit. note 15, pp. 17–32.

29 Hudson, op. cit. note 7.

30 Hudson, W. D. (1969). Editor's introduction: The ‘is‐ought’ problem. In W. D. Hudson (Ed.), *The Is/Ought question. A collection of papers on the central problem in moral philosophy* (pp. 11–31). MacMillan and Co LTD.

31 Ayer, A.J. (1946). *Language, truth and logic*. Dover Publications Inc.

32 Pigden, C. R. (1989). Logic and the autonomy of ethics. *Australasian Journal of Philosophy*, *67*(2), 127–151. https://doi.org/10.1080/00048408912343731, p. 130.

33 Hudson, op. cit. note 30, pp. 13–14.

34 (2)(a) did not have much representation even in the volume by Hudson.

35 Brody, op. cit. note 5, p. 211.

36 This article follows this recipe too.

37 Harris, op. cit. note 9, pp. 18–27.

38 Ayer, A. J. (1946). Critique of ethics and theology. In *Language, truth and logic* (pp. 102–120). Dover Publications Inc.

39 Ayer, op. cit. note 31.

40 Hare, op. cit. note 24.

41 Black's essay is illuminating: “A second reason that may predispose modern philosophers to accept Hume's Guillotine is the view, more popular in our own time than in his, that ‘ought’‐statements make no truth claims at all and are therefore disqualified to serve either as premises or as conclusions,” Black, op. cit. note 19, p. 102.

42 Hare, R. M. (1993). *Essays on bioethics*. Clarendon Press.

43 Pigden, op. ci*t*. note 32, p. 130.

44 McMillan (2016), op. cit. note 15, pp. 17–32.

45 Unless one supports Hare's universal prescriptivism, or any other theory that does *not* claim that ethics is a form of knowledge.

46 Jonsen, A. R. (1998). The philosophers: Clarifying the concepts. In *The birth of bioethics* (pp. 65–89). Oxford University Press.

47 Potter, V. R. (1970). Bioethics, the science of survival. *Perspectives in Biology and Medicine*, *14*(1), 127–153.

